# Transcriptome profiling of liver of non-genetic low birth weight and long term health consequences

**DOI:** 10.1186/1471-2164-15-327

**Published:** 2014-05-01

**Authors:** Alberto Miranda, Angela P López-Cardona, Ricardo Laguna-Barraza, Alexandra Calle, Irene López-Vidriero, Belén Pintado, Alfonso Gutiérrez-Adán

**Affiliations:** 1 Dpto. de Reproducción Animal, INIA, Avda Puerta de Hierro no. 12, Local 10, Madrid 28040, Spain; 2 Servicio de Genómica, CNB-CSIC, Universidad Autónoma de Madrid, Madrid, Spain; 3 Servicio de Transgénesis, CNB-CBMSO CSIC, Universidad Autónoma de Madrid, Madrid, Spain; 4 G.I. Biogénesis, Universidad de Antioquia, Antioquia, Colombia

**Keywords:** Microsomia, Epigenetic, Metabolic syndrome, Liver, Microarray

## Abstract

**Background:**

It is believed that the main factors of low prenatal growth in mammals are genetic and environmental. We used isogenic mice maintained in standard conditions to analyze how natural non-genetic microsomia (low birth weight) is produced in inbred mice and its long term effect on health. To better understand the molecular basis of non-genetic microsomia, we undertook transcriptome profiling of both male and female livers from small and normal size mice at birth.

**Results:**

Naturally occurring neonatal microsomia was defined as a gender-specific weanling weight under the 10th percentile of the colony. Birth weight variation was similar in inbred and outbred lines. Mice were phenotyped by weight, size, blood pressure, organ size, their response to a glucose challenge, and survival rates. Regardless of diet, adult mice born with microsomia showed a significantly lower body weight and size, and differences in the weight of several organs of microsomic adult mice compared to normal birth weight adults were found. After a high-fat diet, microsomic mice were less prone to obesity, showing a better glucose tolerance and lower blood pressure. Through a transcriptome analysis, we detected a different pattern of mRNA transcription in the liver at birth comparing male vs female and microsomic vs normal mice, noting some modifications in epigenetic regulatory genes in females and modifications in some growth factor genes in males. Finally, using embryo transfer of embryos of different quality and age, we identified a putative preimplantation origin of this non-genetic microsomia.

**Conclusions:**

(1) neonatal microsomia is not always a risk factor for adult metabolic syndrome, (2) neonatal non-genetic microsomia displays changes in the expression of important epigenetic genes and changes in liver mRNA transcription profile at birth, exaggerating sexual dimorphism, and (3) random preimplantation phenotypic variability could partially explain body birth weight variation in isogenic lines.

## Background

Human Intrauterine Growth Restriction (IUGR) causes a reduction of fetal growth rate and frequently derives into low birth weight [1]. Worldwide, more than 20 million children suffer this last syndrome, representing a global prevalence of 15.5%, from which 95% occurs in low and middle income countries (LMICs). Birth weight is widely accepted as a prognosis factor of fetal and neonatal health [2] but although not all low values for this feature are pathological, it is mostly associated to IUGR in LMICs [3]. As a consequence, it seems to produce a higher risk of neonatal and perinatal mortality, aging metabolic diseases and neurocognitive disorders [4-6].

In most polytocous species, each newborn in a litter will differ in size and weight. However, random variation in these measurable biological traits is not remarkably diminished by reducing genetic variation using inbred strains or reducing environmental variability through standardized husbandry in laboratory animals. It is propose that processes during early embryogenesis may cause this intangible variance [7]. In pigs, litter size, parity, age of sow and season at conception were observed to only explain 20% of within-litter variation in piglets birth weight, meanwhile factors such as embryo genotype or epigenetics that affect embryo and fetus development would be responsible of most of heterogeneity [8]. Moreover, pathological low birth weight has been correlated with a risk of metabolic syndrome (diabetes, hypertension and obesity in adulthood) [(Developmental Origins of Health and Disease (DOHaD)] [9-13] and most DOHaD studies have used birth weight as an entry criterion to explore its long term effects. However, to date there is scarce evidence to suggest that naturally (non-genetic) occurring low birth weight may have a negative long term effect, and we lack data on the subsequent characteristics developed by each sex.

In the current study, we examined natural birth weight variations in inbred mice (homozygous at every allele) to determine how a single genotype in a similar environment may give rise to different phenotypes at birth. Microsomia was concretely assessed, which is present in those individuals with weight at birth below the 10^th^ percentile of the colony. We also analyzed the mechanisms that give rise to the phenotypic changes of these naturally produced small mice and compared the transcriptional profile of the liver at birth in normal, microsomic (M) male and M female mice. Any observed phenotypic differences among these inbred mice are mostly epigenetic because, by definition, genetic variation has been eliminated and environmental changes have been reduced. Although the underlying mechanisms of fetal programing are still unknown, epigenetics has been suggested as one of the possible explanations for links observed between intrauterine risk factors and metabolic syndrome development. The mechanisms whereby an event that occurs early in life may have long term effects on the phenotype of an organism many years later are only now starting to emerge [14-17]. Understanding such processes will help to develop preventive and treatment strategies for common diseases such as type 2-diabetes or cardiovascular disease.

## Results

### Microsomic growth adaptation in newborn inbred mice

**Figure 1:**
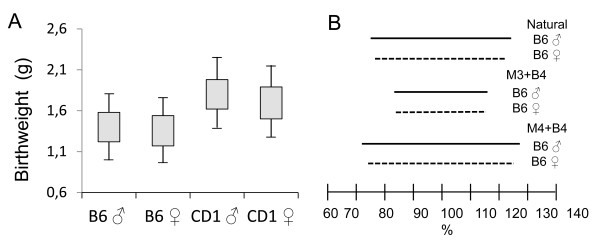
**No differences were observed in birth weight variation in male and female CD1 outbred vs B6 inbred mice. (A)** Box-Whisker plot indicating mean ± SD (box) and mean ± 1.96 SD (lines). **(B)** Comparing the full range of random phenotypic variability (4× coefficient of variation) of birth weight in six groups of mice: naturally born male and female B6 mice (top lines), mice born after the simultaneous transfer of five *in vivo* produced Day 3 morulas (M3) and five Day 4 blastocysts (B4) (middle lines) and mice born after the simultaneous transfer of five *in vivo* produced Day 4 morulas (M4) and five Day 4 blastocysts (B4) (bottom lines).

**Figure 2:**
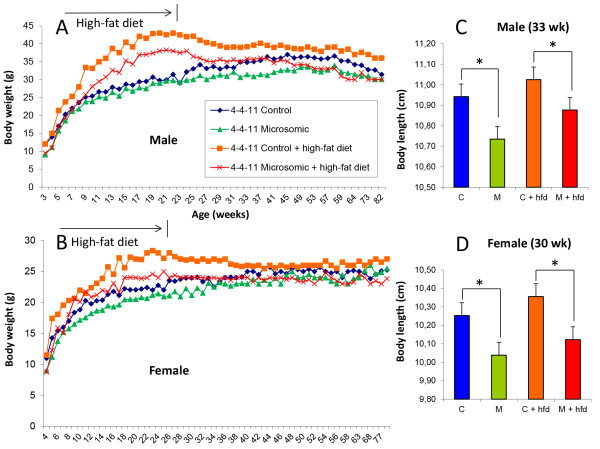
**Microsomic animals are reduced in size and weight and more resistant to obesity than control animals.** Body weights recorded over an 80-week period in M and C males **(A)** and females **(B)** fed a normal or high-fat diet (hfd) for 18 weeks (from weeks 6 to 24 of age). Body lengths recorded for M and C males **(C)** and females **(D)** at 30-33 weeks of age following 18 weeks of a normal or hfd (*P < 0.05. Error bars, s.e.m.).

C57BL/6 N and CD-1 males and females were mated under conditions of standardized husbandry and individuals from the resulting litters were measured at birth. About 10% of the newborn animals were significantly smaller and weighed less (below the 10th percentile for the colony weanling weight) than genetically identical animals in the same litter. This suggests an intrauterine factor-driven growth alteration or the normal consequence of random phenotypic variability. No differences were detected in the mean standard deviations of birth weights recorded for 20 litters of outbred (CD1) versus inbred line (C57BL/6 N) (Figure 1A). At weaning, the M mice also weighed significantly less than their litter siblings (p < 0.01) (Figure 2A and 2B).

**Figure 3:**
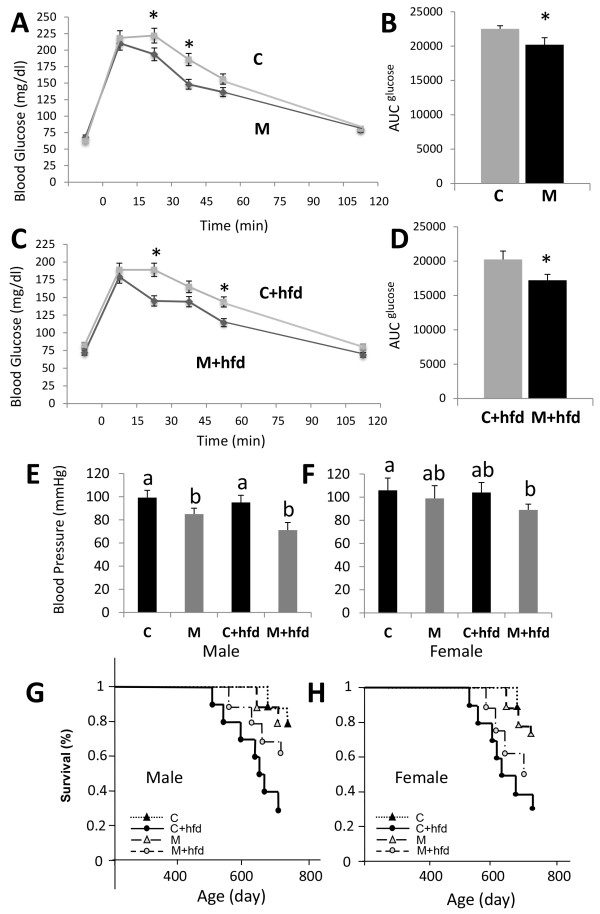
**Microsomic animals recover faster from a glucose challenge and show a lower systolic blood pressure (SBP) than control animals.** Glucose tolerance test performed in M and C male mice fed with a normal **(A)** or a high-fat diet (hfd) for 18 weeks **(C)**. Area under the curve (AUC) for the glucose tolerance test performed in M and C mice fed with normal **(B)** or high-fat diet (hfd) for 18 weeks **(D)** (*P < 0.05. Error bars, s.e.m.). SBP recorded in M and C males **(E)** and females **(F)** fed with a normal or hfd for 18 weeks, respectively. [a, b] indicate a significant difference at *P* < 0.05. Error bars, s.e.m. Survival rate in M and C male **(G)** and female **(H)** fed with or without hfd, respectively.

To examine the phenotypic response to a high-fat diet (hfd), mice were fed a normal diet (C) or a diet rich in lipids from weeks 6 to 24 of age (C + hfd). At the end of this period, significant weight and size differences were observed between the M and the control normal birth weight animals, regardless of sex and diet (p < 0.05) (Figure 2). However, these differences were significantly more pronounced throughout the entire life span of the mice in animals receiving the high-fat diet (p < 0.01). In order to assess anatomical variations derived from both diet and corporal condition (M or C), weight of 90 weeks old mice as well as their organs were recorded (Additional file 1: Table S1). Interestingly, liver, which plays an important role in metabolism and thus in the potential hfd outcome, didn’t show any significant variation between M and C mice for both diets, regardless of sex. Several differences were found in other organs, however they lacked apparent correlation with the considered conditions. These results suggest a metabolic regulation pathway present in the M mice that renders them more resistant to obesity than control mice when given a diet rich in lipids. Moreover, unlike controls, M male mice on high-fat diet for 18 weeks recovered a similar weight to that of M not given a high-fat diet. The lack of catch-up growth in our mice model (Figure 2) indicates the animals had not suffered nutrient restriction since at the time of weaning and well into adulthood, microsomic mice were always lighter in weight and shorter in length.In order to assess the presence of metabolic syndrome, a glucose tolerance test and systolic blood pressure (SBP) measurements were performed at 6 months of age, a life span in which mice are considered to have reached the adult age. When subjected to a glucose tolerance test, M mice recovered normal glucose levels faster than controls (Figure 3A and 3C). Moreover, the area under curve (AUC) was significantly reduced for both diet groups of animals (Figure 3B and 3D), suggesting an adaptive response of glucose levels in M animals. Similar results were recorded in SBP irrespective of diet although differences were greater for M males than M females relative to their respective controls (Figure 3E and F).Both M and C mice were maintained for 2 years to assess the possible appearance of any disorders with a late age of onset. However, no differences in age-related pathologies were seen between C and M mice. Figure 3G and 3H shows survival rates in males and females of control (C) and microsomic (M) mice fed with both normal diet and lipid rich diet during 24 weeks (hfd). Survival rate of C and M animals was similarly high for both genders (range 70-80% at 24 months) while C + hfd mice have a significant decrease in comparison with M + hfd mice. Both groups fed with hfd have a significant decrease on the survival rates in comparison with normal diet.

To determine whether birth weight variation originates during the preimplantation or fetal period, birth weight variations were compared after the simultaneous transfer of *in vivo* produced good quality embryos (IETS grade 1) of different age and developmental stage (50% Day 3 morulas and 50% Day 4 blastocysts) and the simultaneous transfer of *in vivo* produced embryos (without selecting good quality embryos) of the same age (Day 4) but different developmental stage (common in mice) (50% blastocysts and 50% morulas). Birth rates were 81% versus 57% in first and second experiments respectively. Thus, the rate was reduced when embryos of different quality were transferred. After weighing mice from 12 litters for both experiments, we observed that only the transfer of embryos of the same age though different developmental stage gave rise to a higher coefficient of variation [(standard deviation/mean)/100] in body weight at birth (Figure 1B), and that the transfer of selected good quality embryos comprising 3-day-old morulas and 4-day-old blastocysts led to reduced birth weight variations, suggesting the preimplantation origin of this alternative developmental program.

### Microarray analysis: overall results and validation

To evaluate gene expression variations in normal and M animals, a microarray analysis of liver from newborns was performed. Liver was selected since it is one of the relatively more homogeneous organs that can suffer the effects of an abnormal environment [18]. In our case, the effects of a putative developmental malnutrition that could lead to microsomia would have certain transcriptional effects in this organ, since it is an important step in nutrient metabolism and detoxification. Moreover, the absence of significant liver weight variation due to the common fat accumulation in the hfd group supported the possibility of gene expression changes in order to prevent it. Hence, liver is one of the best candidates to assess whether this phenotype has an intrauterine environmental origin. The Mouse Agilent microarray system used covers 39,430 unique genes and 16,241 lincRNAs. Twelve expression datasets (3 independent experiments using liver tissue from normal –control– males/females and M males/females mice respectively) were obtained and the following calculations were performed according to the *GeneSpring GX* (Agilent Technologies) manufacturer’s instructions. After selecting genes according to their P-value (limma; t-test) of significance (P <0.01), these genes were further filtered to remove those whose expression was below a threshold, based on median normalized intensity values, which was considered to be the no-change threshold (fold change >1.5). Four pairwise comparisons were performed: 1) control (C) males vs M males; 2) C females vs M females; 3) C males vs C females; and 4) M males vs M females.

In total, 776 differentially expressed genes (DEGs) were detected for M vs C females, most of which were upregulated in M females (525 vs 251) (Additional file 2: Figure S1). For M vs C males, we detected 437 DEGs, also mostly upregulated in the M group (330 vs 107), suggesting a more complex regulation in females (Additional file 2: Figure S1). Some DEGs were common to M mice groups, such as the epigenetic regulator *Trim28*, which was upregulated in M females while downregulated in M males in comparison to their respective controls. The *Pbx1* and retinoic acid pathway genes (*Ppar*, *Rbp3*, *Crabp*-*II* and *RxR*) were also differentially expressed in the two experimental comparisons, with M animals showing greater expression in both comparisons. Further important DEGs such as *Chd1* and *Ncor1* were specifically upregulated in M males compared to C group and *Hdac5*, *Igf*-*1*, *Tgfb1* and *c*-*Met* were upregulated in M females compared to C mice.

To validate the array results, 11 genes were chosen for RT-PCR analysis in independent samples: three pluripotency related genes (POU5F1, SOX2, NANOG), two trophectodermal markers (KRT18, CDX2), one gene associated with DNA methylation (DNMT3B), one gene involved in polyamine biosynthesis (ODC1), and four genes related to nutrient homeostasis (RGN, STC2, SLC6A6, SLC7A1). No differences were observed between the real time PCR and array results (Table 1).

**Table 1:** Validation of array data by real-time qRT-PCR analysis (expression fold changes)

**Female microsomic vs control**			**Male microsomic vs control**		
**Gene**	**qPCR**	**Array**	**Gene**	**qPCR**	**Array**
Smarcc1	11.32	10.81	Crabp2	13.97	15.05
Ruvbl2	6.30	5.14	Chd1	2.09	1.99
Hdac5	3.65	3.72	Vegfa	-2.34	-1.94
Trim28	2.03	2.35	Fgf23	-2.09	-2.37
Irs2	2.45	2.17			
Gata-1	-2.91	-2.07			
Igf1	-2.60	-2.37			

### Gene ontology

The gene ontology classification system (FatiGo) was used after enrichment score (ES) analysis (ES ≥ 1.3) to classify genes according to biological function and corresponding cell components [19]. For M vs C males, genes involved in translation and steroid metabolic process were upregulated in mice with microsomia while transcription regulating genes were downregulated. Golgi apparatus and ribosomal genes were upregulated while nuclear genes were downregulated (Additional file 3: Figure S2). For M vs C females, genes involved in the processes cell proliferation, transcription regulation, tissue morphogenesis, gland morphogenesis and skeletal system development were upregulated while purine metabolism, pyrimidine metabolism, mRNA processing, M phase cell division and condensed chromosome were downregulated. Nuclear and cytoplasm genes were upregulated while ribonucleoprotein complex, chromosome and non-membrane-bound organelle genes were downregulated (Additional file 4: Figure S3). The results of comparing C males vs C females with M males vs M females are indicated in (Additional file 5: Figure S4 and 6: Figure S5).

## Discussion

In this work, we report an inbred mouse model of naturally occurring low birth weight, of non-genetic origin, and with no detrimental long term effects. The low birth weight produced at parturition is here ascribed to natural preimplantation developmental variability. Interestingly, newborn male and female mice with M increased differentially their weight in response to a high-fat diet. This particular feature of M mice contrasts with the long term negative effects of a low birth weight observed in other studies on small animals addressing the DOHaD theory [20-22]. In addition, we noted that M animals receiving a high-fat diet were more resistant to acquiring an overweight phenotype and have higher survival rates than controls. Curiously, liver didn’t undergo weight changes due to fat accumulation, a feature which is relatively common in certain types of hfd [23-26]. Consequently, several transcriptional alterations could be happening that could explain this feature. Moreover, the different metabolic programs detected with respect to genetically identical littermates suggest epigenetic alterations in these M animals. Differential total weight gain was also observed when considering sex, indicating a divergent physiological mechanism between males and females [13,27-29]. This observation was confirmed by gene ontology analysis. Indeed, in M males, only a few post-transcriptional modifications and enrichment in metabolic genes and the corresponding organelles (ribosomes and Golgi apparatus) were observed, suggesting protein-based adaptation. In contrast, a larger number of processes were overrepresented in M females, highlighted by nuclear gene enrichment and the downregulation of chromosome condensation.

To better understand the molecular basis of non-genetic microsomia, a microarray experiment was conducted on the liver of newborns. Microsomic females showed a significantly higher number of DEGs than M males compared with their respective controls (776 vs 437), supporting more complex metabolic changes in females. Among the DEGs detected, the important epigenetic regulator *Trim28* (tripartite motif-containing 28) was found in both sexes. Also known as *Kap1* (KRAB-associated protein-1), the gene *Trim28* is ubiquitously expressed throughout development [30] and is a critical regulator of this process. Some authors have attributed to this protein an important role in epigenetic control whereby it physically attaches itself to several histone methyltransferases (HMTs) [31], synergistically triggering the methylation of transposons [32]. In MF, *Trim28* was upregulated along with *Hdac5* (Additional file 7: Table S2) compared to CF, which suggests a change in the epigenome as the mechanism for the alternative metabolic program. This observation is consistent with the findings reported by another study in which epigenetic changes in histones induced by a *Trim28* heterozygous deletion were linked to weight variations at birth [33]. This latter study also revealed a high predisposition of female *Trim28* heterozygous mice to impaired glucose tolerance. Moreover, we have observed in a previous work persistent epigenetic changes and glucose intolerance phenotypes in animals born after embryo *in vitro* culture, including M animals showing improved glucose tolerance over controls [34]. A more efficient use of glucose could therefore explain why the M animals in the present study weighed less during the whole experiment, regardless of sex or diet.

**Figure 4:**
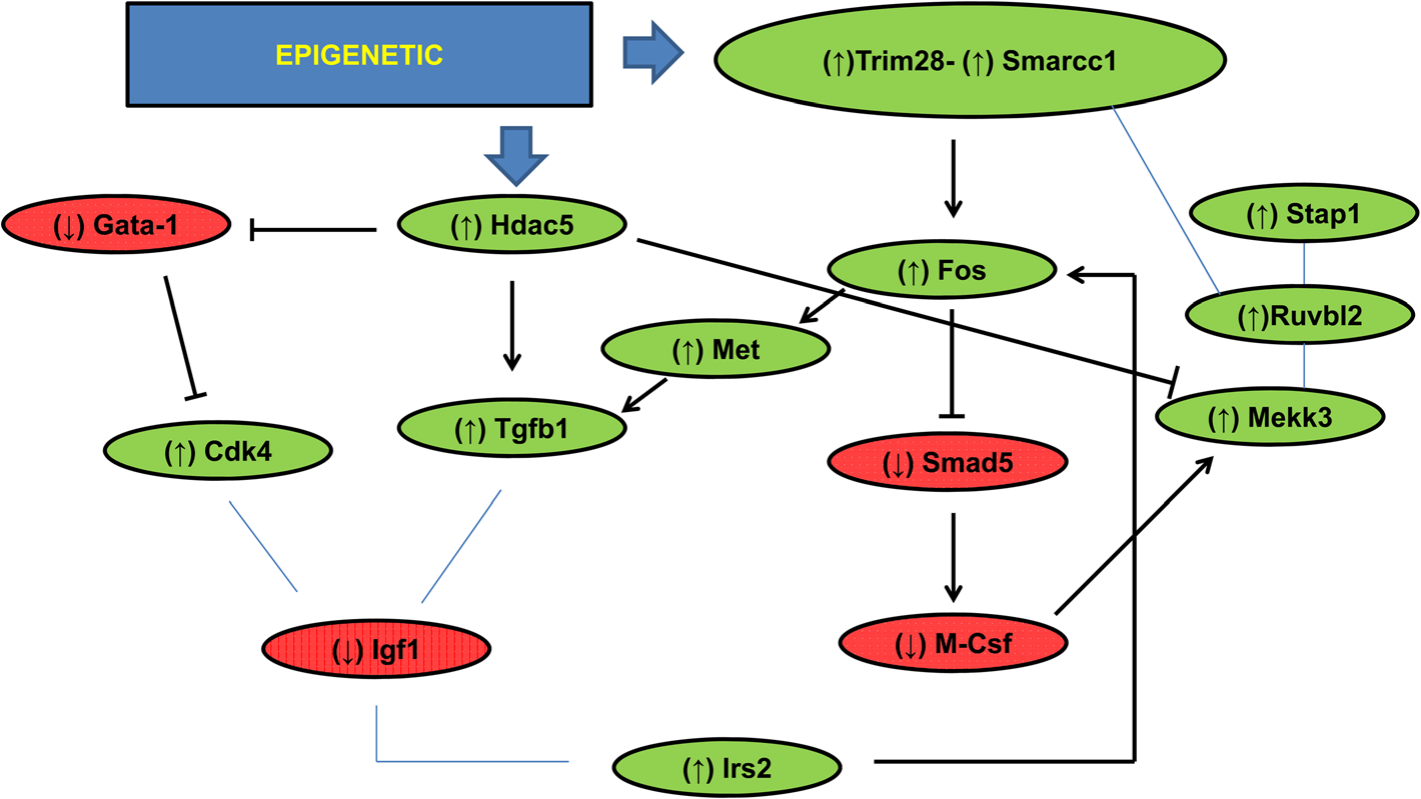
**Suggested model and summary of epigenetic changes possibly occurring in MF vs CF.** Green circles: upregulated genes; Red circles: Downregulated genes (: activation; : inhibition; : protein complex).

Ingenuity Pathway Analysis of deregulated genes revealed that epigenetic-mediated *Trim28* upregulation in MF mice could lead to the upregulation of several markers that could explain this phenotype (Figure 4) such as *Igf*-*1* (insulin growth factor-1) and *c*-*Met* proto-oncogene. *Igf*-*1* has been identified as an important factor for developmental growth [35]. Our results for MF indicated the overexpression of this gene in offspring compared with controls (Additional file 7: Table S2). These data are in line with published results, whereby elevated hepatic *Igf*-*1* mRNA and plasma IGF-1 levels were attributed to an attempt to compensate for a reduced hepatic and body size at birth [36]. Moreover, this marker has been also related to epigenetic modifications in H3K4 histone [36], thereby supporting our hypothesis of this type of modification in our model.

The proto-oncogene c-MET is a transmembrane tyrosine kinase cell surface receptor for hepatocyte growth factor (HGF), which is mainly involved in cell proliferation and tumor expansion [37,38]. In the liver, c-MET regulates a large number of molecular pathways related to its plasticity [39], thus the high expression levels observed for this gene in MF (Additional file 7: Table S2) could be attributed to an adaptation process. This finding together with the fact that no *c*-*Met* transcription variation was detected in microsomic males, could support the idea of a more complex adaptation process in females. Importantly, this gene has also been associated with skeletal muscle atrophy in mice [40], providing an alternative explanation for the low weight shown by these animals at birth. In addition, c-MET has been recently related to the capacity of the liver to regulate blood glucose levels, inducing hepatic uptake and inhibiting output [41]. Thus, the lower AUC obtained in the glucose tolerance test could be explained by the synergistic reduction of blood glucose and IGF-1 levels.

Curiously and contrary to the case in females, *Trim28* was downregulated in M males compared with Cs (Additional file 7: Table S2). *Chd1* (chromatin remodeling factor-1) was also found to be upregulated as were other components of this epigenetic protein complex, namely *Ncor1* (nuclear receptor corepressor-1) and *Pbx1* (pre B-cell leukemia homeobox-1) (Additional file 7: Table S2). CHD1 is a helicase DNA binding protein required to maintain the open chromatin of pluripotent mouse embryonic stem cells [42]. This protein associates with the promoters of active genes to maintain the current transcriptional profile [42] in response to epigenetic changes such as methylations [43]. In the same line, NCoR1 co-repressor protein has been identified in histone-deacetylase (HDAC) complexes with a role in chromatin structure modulation [44], and PBX1 has been described to regulate the activity of the epigenetic coactivators HDACs and histone acetyltransferases [45]. These data again support the hypothesis of epigenetic alterations provoking the altered gene expression profile of microsomic animals.

**Figure 5:**
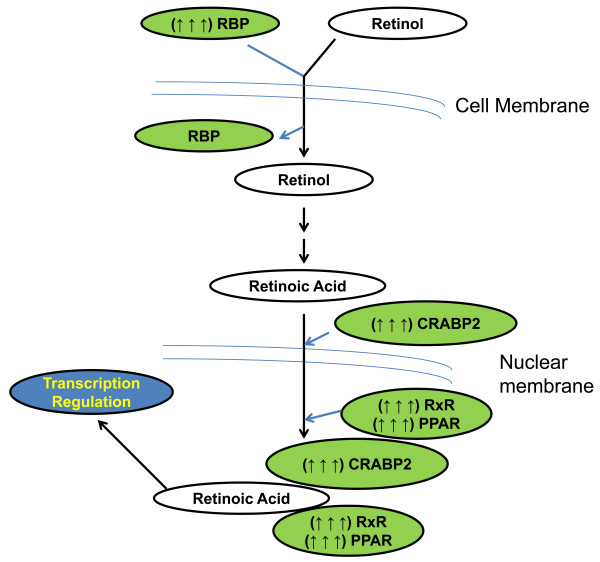
**Suggested model and summary of expression changes in the RA pathway possibly occurring in microsomic animals.** Green circles: upregulated genes; White circles: No change in expression (: activation.

Ingenuity Pathway Analysis of deregulated genes indicated that some members of the retinoic acid (RA) canonical pathway were upregulated in microsomic animals of both sexes (Additional file 7: Table S2) (Figure 5) in comparison to their respective controls (C male and C female). Several early studies revealed that nuclear receptors such as retinoid X receptors (RXR) and thyroid hormone receptor (TR), translocate to the nucleus and associate with nuclear corepressors such as NCoR1 or NCoR2 (or SMRT, silencing mediator for retinoid and thyroid hormone receptors) [46,47]. As mentioned before, these nuclear receptors recruit HDAC, resulting in histone deacetylation, chromatin compaction, and silencing of target gene expression [44,48]. Hence, in the case of M males, the RA pathway could be mediating the epigenetic changes needed for the correct adaptation to intrauterine environment variations in collaboration with the upregulated NCoR1. Interestingly, numerous research efforts have revealed a feed-forward mechanism of RA synthesis mediated by HOX-PBX1 during certain stages of development [49]. Although interactions of the HOX-PBX1 system with DNA were found to be dispensable for RA to control the transcription profile [50], a possible role of PBX1 in regulating imprinted genes is supported by the present findings. Thus, in our model of M males, it could be that PBX1 enhances the activity of the RA pathway by increasing RA synthesis, triggering as a consequence some epigenetic changes in cooperation with NCor1.

Recent data suggest that *Igf1* expression is promoted by all-trans retinoic acids (ATRA) in epidermal and skeletal tissue [51]. In this latter study, variations in the developmental gene profile were detected in response to a high-vitamin A (retinol) diet in fish larvae. As a result, body size and weight were significantly reduced, and *Tgfb1* and *RxR* were upregulated, along with *Igf1* in later stages of development [51]. In addition, ATRA have a growth inhibitory effect through upregulation of the *c*-*Met* gene [52]. Since we observed a similar transcription profile (Additional file 7: Table S2) and phenotype in our MF model, it could be suggested that epigenetic variations arising from the RA pathway, among others, induced a different gene expression profile and a reduction in body weight and size. Remarkably, the findings of other studies have shown how RA and upregulation of the components of the CRABP-II/RA pathway [53] prevent diet-induced weight gain in mice by inhibiting adipogenesis *in vivo*[54]. Related to this, other investigations have determined that besides preventing induced obesity ATRA also improve glucose intolerance and high SBP. These transcriptional features mimic what happens in our model, interestingly, independent of sex (Additional file 7: Table S2) (Figure 5). Certainly, without considering the influence of diet, normal, or even improved, levels of glucose tolerance and SBP were obtained in M animals. Although several other DEGs in these animals could contribute to this behavior, they could all be the consequence of subsequent global modifications derived from the main RA branch.

## Conclusions

The concept of early life programing is widely accepted, but for a complete picture of DOHaD we have to understand the different mechanisms of adaptation. Indeed, our work reveals that a low birth weight is not always an indicator of intrauterine nutrient restriction, and that it could be the consequence of stochastic epigenetic variation. Numerous studies have consistently found evidence of this stochastic epigenetic variation as the driving force for developmental and evolutionary adaptation [55-59]. Thus, raised in an identical environment, many loci with highly variable DNA-methylated regions have been identified in the liver and brain of newborn isogenic mice, as well as in the human liver. These loci are associated with development and morphogenesis, and with the functional property of expression. Correspondingly, it has been reported that a range of random variability in quantitative biological traits, for example body weight at 3 months, may originate from early embryo development [7]. Here we confirm that phenotypic variation in preimplantation embryos could be the origin of the random variability of certain measurable biological traits, such as the natural variation in birth weight observed in our inbred mice. We hypothesize that, in early embryos, phenotypic variation is a strategy aimed at maintaining fitness (reproductive success) in a changing environment. This type of adaptive response or plasticity is called anticipatory or predictive adaptation as opposed to immediate adaptation (occurring in the detrimental Intrauterine Growth Restriction [IUGR] adaptive response in the fetus) and comes into play because the early preimplantation embryo lacks the time, organs and/or physiological tools to immediately adjust its phenotypic development to a new challenging environment. Sex differences detected between early embryos could also be related to sex differences in microsomic adaptation [60,61]. Moreover, we describe here a microsomic epigenetic model based on natural embryo variability that is able to avoid diabetes and/or metabolic syndrome. Upregulation of the RA pathway (including *Crabp*-*II*, *Ppar*, *Rbp* and *RxR*) together with a putative increase in the synthesis of its main agonist (RA) via the HOX-PBX1 complex, suggest an epigenetic regulation mechanism whereby abnormal blood glucose levels, high SBP and obesity could be prevented or reversed. Hence, it becomes clear that mammalian early embryos undergo large scale reprograming of DNA methylation patterns with the potential to create epigenetic diversity among cells and individuals [62]. This could be important for cell fate decisions in development and for creating phenotypic diversity in early embryo development.

## Methods

### Animal model

All experimental procedures involving mice were approved by the Ethics Board of the INIA and performed according to the Guide for the Care and Use of Laboratory Animals adopted by the Society for the Study of Reproduction and to European legislation. Mice were fed a standard diet (Global Diet 2014, Fat 13%, Protein 20% and Carbohydrates 67%; Harland Iberica) or high fat diet (from week 6 to 24 of age) (TD.08811 45% kcal Fat Diet (Fat 44,6%, Protein 14,8% and Carbohydrates 40,6%); Harland Iberia) ad libitum and kept in a temperature- and light-controlled environment (22–24°C, 14 L:10D). Adult C57Bl/6 N mice (Harland) were bred for 2 weeks, and then transferred to individual cages. Dams delivered naturally, fostered their own pups, and only litters with 6 to 8 animals were used in the experiments. Pups were weighed one day after birth and upon weaning (Day 20 of life).

Thereafter, weights were recorded each week. Utilizing standard clinical criteria, microsomia was identified by a gender-specific weight below the 10th percentile at birth. For control mice, appropriate growth was defined as a weight within 1 standard deviation of the colony mean. For each microsomic mouse detected (36 males and 38 females), the corresponding littermate control mouse was identified (37 males and 35 females). All study groups consisted of mice from at least 14 different litters. ICR (CD-1®) Outbred Mice (Harlan) were used to quantify birth weight variability in an outbred mouse line. Growth conditions were similar to those used for the C57BL/6 N mice, but in this case only litters with 7 to 10 animals were used in the experiment. To examine the phenotypic response to a high-fat diet, mice were fed a normal diet or a diet rich in lipids from weeks 6 to 24 of age. For the organ weight, two year old mice were sacrificed and some viscera, including the liver, lung, heart, kidney, spleen, and testes were excised and accurately weighed. The coefficients of these organs to body weight were calculated as the ratio of tissues (wet weight) to body weight.

For experiments of embryo transfer, embryos were produced using female C57BL/6 N mice aged 6–8 weeks old. Superovulation was induced with an intraperitoneal injection of 7.5 IU equine chorionic gonadotropin (eCG, Folligon, Intervet, Spain), followed 48 h later, by 5 IU of human chorionic gonadotropin (hCG). Immediately after hCG administration, females were paired with a male of the same genetic background in an individual cage overnight. Morula and blastocysts were collected from the uterus of females 3 or 4 days after mating. Embryos (10 to 15 per female) were transferred into the uterus of Day 3.5 C57BL/6 N pseudopregnant females [63].

### Baseline studies

At 6 months of age, glucose tolerance tests were performed using 10 animals per group. Mice were transferred to a clean cage and left to fast overnight (16 h) with ad libitum access to water. The following morning, the animals underwent a blood glucose tolerance test. Whole-blood b-D-glucose levels were determined using a standard handheld glucometer (Glucocard Gsensor, Arkray Factory, Inc.) on blood samples (2 μl/measurement) collected from the tip of the tail. Following baseline glucose measurements, the mice were injected i.p. with glucose (20% solution, 1.5 mg/g). Blood glucose readings were then taken at 15, 30, 45, 60, and 120 min postinjection. Food was supplied immediately following the last measurement. The area under the curve (AUC) was calculated for plasma glucose (AUCglucose) levels for the entire 120-min study period according to the trapezoid rule.

For blood pressure measurements, 6 months age mice were acclimated to restraint and tail-cuff inflation. The restraint platform was kept at 33–34°C. The mouse was placed in a metal box restraint with its tail passing through the optical sensor and compression cuff and taped to the platform. A traditional tail-cuff occluder was placed proximally on the mouse’s tail, and the animal was then immobilized by taping to a V-shaped block between a light source (above) and a photoresistor (below). On inflation, the occluder stops blood flow through the tail, and on deflation return blood flow (RTF) is detected by the sensor. Amplifier and instrument controls were set during an initial series of inflation-deflation cycles. This instrument (model BP 2000 Blood Pressure Analysis System, Visitech Systems; Apex, NC) automatically takes 10 30-s measurements using proprietary software (BP-2000 Software Beta Version 03/10/97). If at least 5 out of 10 readings were acceptable, the highest and lowest readings were discarded, and the remaining readings were averaged as a single session value. Ten animals per experimental group were used.

Survival curves were constructed for each group of mice (using the number of animals per group indicated above) fed or not fed with hfd, according to the Dinse-Lagakos method [64].

Body length, assessed by the mean of two measurements of nose-to-tail base distance (with five days of interval), was measured in anaesthetized adult animals at 30 and 33 weeks of age (for female and male respectively). Body lengths were recorded for 20 animals per group.

### RNA extraction

Livers from each experimental group (normal birth weight or microsomal littermates) were extracted from newborn mice. Total RNA was prepared using the *TRIzol*® *Reagent* Kit (Invitrogen) following the manufacturer’s instructions. Samples were additionally treated with *RNeasycolumn* purification kit (QIAGEN) in order to completely remove genomic DNA. Immediately after extraction, total RNA was cooled to -80 ºC until further use.

### Independent verification of array data by real-time RT-PCR

The RT reaction was carried out according to the manufacturer’s instructions (Promega, Madrid, Spain) on 2 μl of total RNA to generate cDNA. Tubes were heated to 70°C for 5 min to denature the secondary RNA structure and then the RT mix was completed by the addition of 5 units of Superscript RT enzyme. After incubation at room temperature for 10 min and then at 42°C for 60 min to allow the reverse transcription of RNA, the mix was heated at 70°C for 10 min to denature the RT enzyme. We used 2 μl of cDNA sample in the RT-PCR mix to detect each transcript.

All mRNA transcripts were quantified by real-time qRT-PCR. At least, three replicate PCR experiments were conducted for all genes of interest. In each sample, relative levels of each transcript and histone H2az were compared. PCR was performed by adding a 2-μl aliquot of each sample to the PCR mix (*Quantimix Easy Sig Kit*, Biotools) containing the specific primers. Primer sequences, annealing temperatures, and the approximate sizes of the amplified fragments of all transcripts are shown in (Additional file 8: Table S3). The comparative cycle threshold (CT) method was used to quantify expression levels. Quantification was normalized to the endogenous control H2az. Fluorescence was acquired in each cycle to determine the threshold cycle or the cycle during the log-linear phase of the reaction at which fluorescence increased above background levels for each sample. Within this region of the amplification curve, a difference of one cycle is equivalent to doubling of the amplified PCR product. According to the comparative CT method, the CT value was determined by subtracting the H2az CT value for each sample from the CT value for each gene. To calculate CT, the highest sample CT value was used (i.e., the sample with the lowest target expression) as an arbitrary constant to subtract from all other CT sample values. Fold changes in relative gene expression of the target were determined using the formula 2^–CT^.

### Statistical analysis

Data processing and statistical tests were performed using the SigmaStat (Jandel Scientific) package. Differences in body weight, animal size, glucose levels (in the glucose tolerance test) and gene transcription were analyzed by one-way repeated-measures analysis of variance (ANOVA). When necessary, percentages and proportions were arcsine-transformed to normalize data. When main effects were detected, Holm-Sidak post hoc tests were used for comparisons between groups. The trapezoidal rule was used to determine the AUC of the glucose curves. Mean differences were assessed by one-way repeated-measures ANOVA and significance determined using the Holm-Sidak post hoc test. Significance was set at P ≤0.05.

### Microarray analysis

RNA from the liver of newborn mice was purified according to standard methods (SI M & M). Samples were amplified, labeled using the *Agilent Quick Amp labeling* kit, and hybridized using the Agilent *SurePrint G3 Mouse GE 8x60K Microarray* (*G4852A*-*028005*) (Agilent Technologies, Palo Alto, CA) and Agilent *SureHyb* hybridization chambers. Three independent replicate experiments were performed for each group. The resulting working gene lists of transcripts were imported to the *Ingenuity*® *iReport* microarray analysis program (Ingenuity® Systems) and FatiGo for gene ontology [19].

### Availability of supporting data

Raw data from microarray experiments was submitted to the Gene Expression Omnibus database (http://www.ncbi.nlm.nih.gov/geo). The platform ID is GPL13912 and accession ID is GSE49876.

## Abbreviations

C: Control; DEG: Differentially expressed genes; DOHaD: Developmental Origins of Health and Disease; ES: Enrichment score; HMT: Histone methyltransferase; HDAC: Histone deacetylase; IUGR: Intrauterine growth restriction; M: Microsomic; RA: Retinoic acid; RXR: Retinoid X receptors.

## Competing interests

The authors declare that they have no competing interests.

## Authors contributions

AM: Design experiments, performed research, analyzed data, drafted the manuscript. ALC: Design experiments, performed research, analyzed data. RLB: Performed research, analyzed data. AC: Performed research, analyzed data. ILV: Contributed new reagents/analytic tools, analyzed data. BP: Contributed new reagents/analytic tools, analyzed data. AGA: Design experiments, performed research, analyzed data, drafted the manuscript. All authors read and approved the final manuscript.

## Supplementary Material

Additional file 1: Table S1 Relative organ weights of 90 weeks-old mice (tissue weight/animal weight ×100). Click here for file

Additional file 2: Figure S1 Number of differentially expressed genes (DEGs) in control, microsomic and microsomic vs control animals of both sexes. Red arrows: upregulated genes; Green arrows: downregulated genes; (A) Venn diagram of MM vs MF and CM vs CF mice (B) Venn diagram of MM vs CM and MF vs CF mice. Click here for file

Additional file 3: Figure S2 FatiGo functional analysis of MM vs CM. Click here for file

Additional file 4: Figure S3 FatiGo functional analysis of MF vs CF. Click here for file

Additional file 5: Figure S4 FatiGo functional analysis of CM vs CF. Click here for file

Additional file 6: Figure S5 FatiGo functional analysis of MM vs MF. Click here for file

Additional file 7: Table S2 Principal genes down- and up-regulated in the microsomic vs control comparison for both sexes. Click here for file

Additional file 8: Table S3 Primers used for RT-PCR. Click here for file
