# Supplementary material for: Transcriptome profiling of liver of non-genetic low birth weight and long term health consequences
Source: BMC Genomics. 2014 May 1;15:327. doi: 10.1186/1471-2164-15-327 (PMC4229907; doi:10.1186/1471-2164-15-327)
Supplement: Additional file 1: Table S1 — Relative organ weights of 90 weeks-old mice (tissue weight/animal weight ×100). [file 1471-2164-15-327-S1.docx]

Additional file: Table S1. Relative organ weights of 90 weeks-old mice (tissue weight/animal weight x100)

| **MALE** | **ANIMAL WEIGHT** | **HEART** | **LIVER** | **TESTIS** | **SPLEEN** | **KIDNEYS** | **LUNGS** |
| --- | --- | --- | --- | --- | --- | --- | --- |
| **CONTROL** | **33.16 ± 0.8ab** | **0.65 ± 0.04a** | **4.91 ± 0.2** | **0.65 ± 0.02a** | **0.62 ± 0.12a** | **1.05 ± 0.03** | **0.80 ± 0.08a** |
| **LOW WEIGHT** | **30.35 ± 0.94a** | **0.67 ± 0.02a** | **5.05 ± 0.16** | **0.66 ± 0.02a** | **0.47 ± 0.06b** | **1.01 ± 0.06** | **0.70 ± 0.04a** |
| **CONTROL-FAT DIET** | **35.74 ± 1.105b** | **0.53 ± 0.06b** | **4.42 ± 0.4** | **0.56 ± 0.06b** | **0.57 ± 0.13ab** | **0.95 ± 0.10** | **0.57 ± 0.07b** |
| **LOW WEIGHT- FAT DIET** | **29.92 ± 1.16a** | **0.70 ± 0.04a** | **4.80 ± 0.15** | **0.66 ± 0.02a** | **0.51 ± 0.08b** | **1.12 ± 0.05** | **0.66 ± 0.03ab** |

| **FEMALE** | **ANIMAL WEIGHT** | **HEART** | **LIVER** | **SPLEEN** | **KIDNEYS** | **PANCREAS** | **LUNGS** |
| --- | --- | --- | --- | --- | --- | --- | --- |
| **CONTROL** | **25.26 ± 1.1ab** | **0.62 ± 0.03a** | **4.66 ± 0.13** | **0.74 ± 0.1ab** | **1.07 ± 0.03** | **0.62 ± 0.06ab** | **0.89 ± 0.05ab** |
| **LOW WEIGTH** | **23.92 ± 0.59b** | **0.62 ± 0.03a** | **5.67 ± 0.90** | **0.57 ± 0.08a** | **1.10 ± 0.04** | **0.92 ± 0.07a** | **0.78 ± 0.04a** |
| **CONTROL-FAT DIET** | **27.16 ± 0.82a** | **0.55 ± 0.03b** | **5.26 ± 0.48** | **0.81 ± 0.23ab** | **1.10 ± 0.04** | **0.57 ± 0.06b** | **0.86 ± 0.05ab** |
| **LOW WEIGHT- FAT DIET** | **24.37 ± 0.93ab** | **0.65 ± 0.04a** | **4.56 ± 0.35** | **0.89 ± 0.20b** | **1.13 ± 0.08** | **0.61 ± 0.06ab** | **0.95 ± 0.08b** |

Data are mean ± SD. Letters indicates significant difference (P≤ 0.05). Statistics used Student’s t test for independent samples.
