# Supplementary figures and images for: Transcriptome profiling of liver of non-genetic low birth weight and long term health consequences
Source: BMC Genomics. 2014 May 1;15:327. doi: 10.1186/1471-2164-15-327 (PMC4229907; doi:10.1186/1471-2164-15-327)

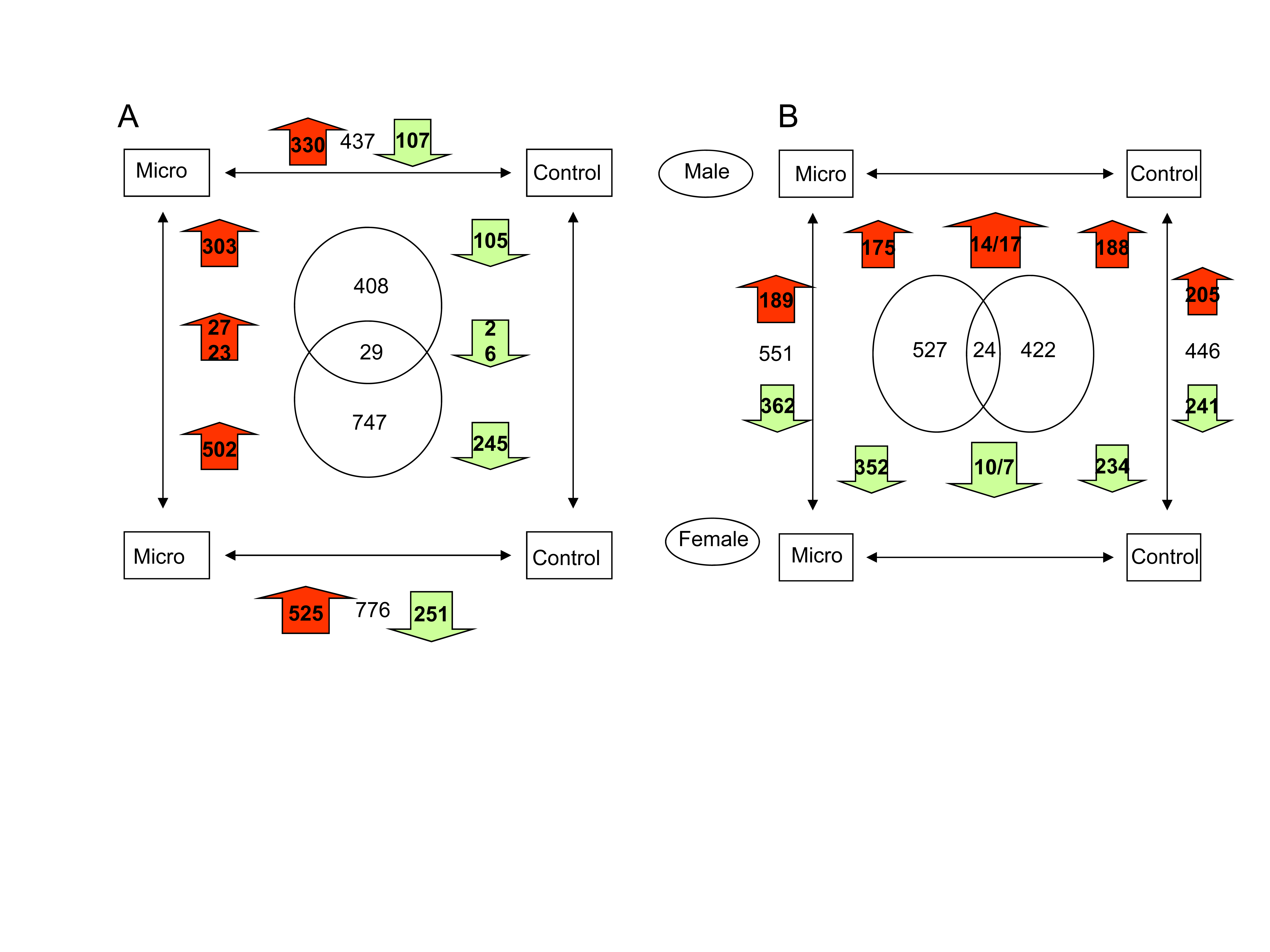

Supplement: Additional file 2: Figure S1 — Number of differentially expressed genes (DEGs) in control, microsomic and microsomic vs control animals of both sexes. Red arrows: upregulated genes; Green arrows: downregulated genes; (A) Venn diagram of MM vs MF and CM vs CF mice (B) Venn diagram of MM vs CM and MF vs CF mice. [file 1471-2164-15-327-S2.tiff]

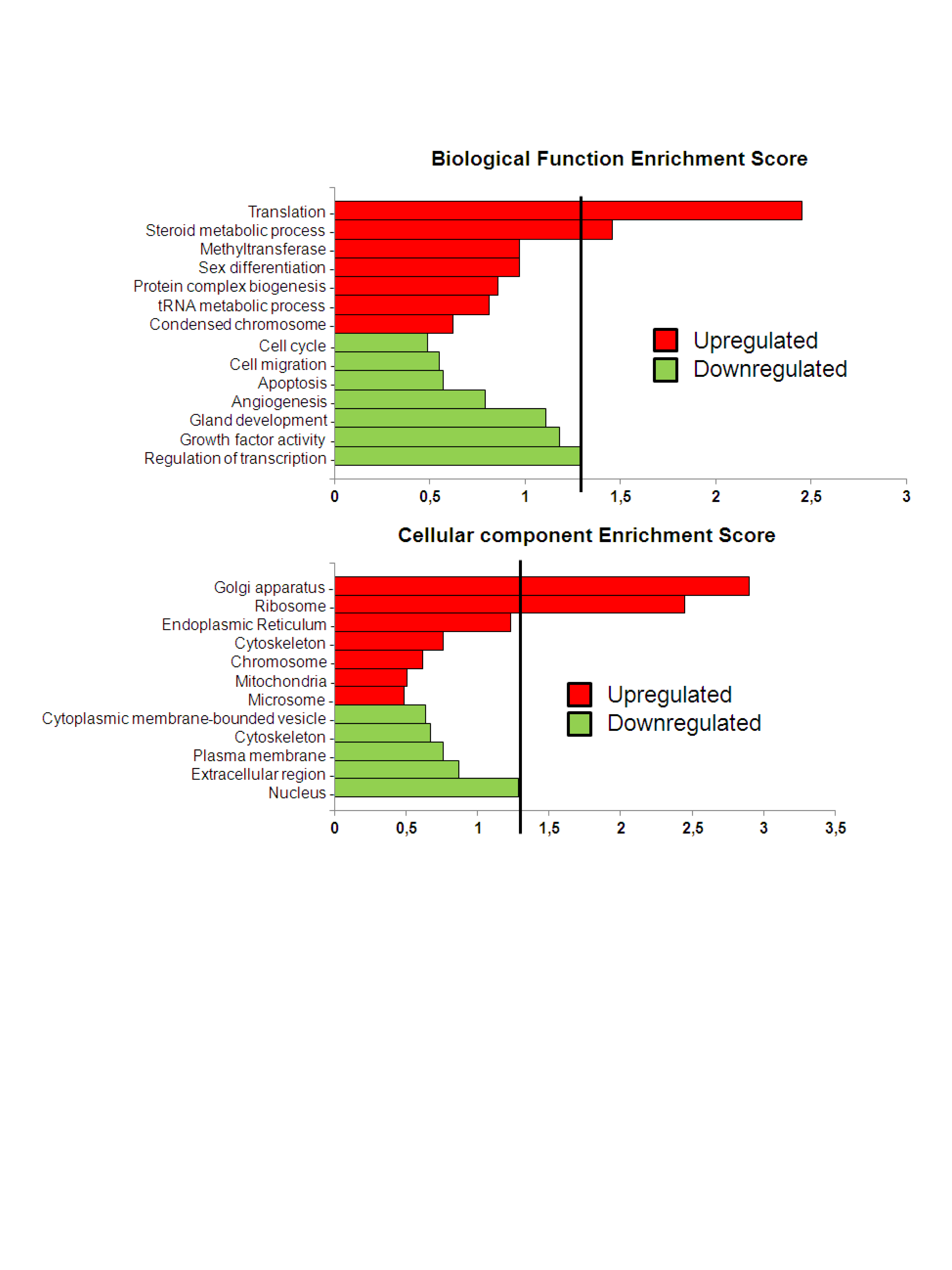

Supplement: Additional file 3: Figure S2 — FatiGo functional analysis of MM vs CM. [file 1471-2164-15-327-S3.tiff]

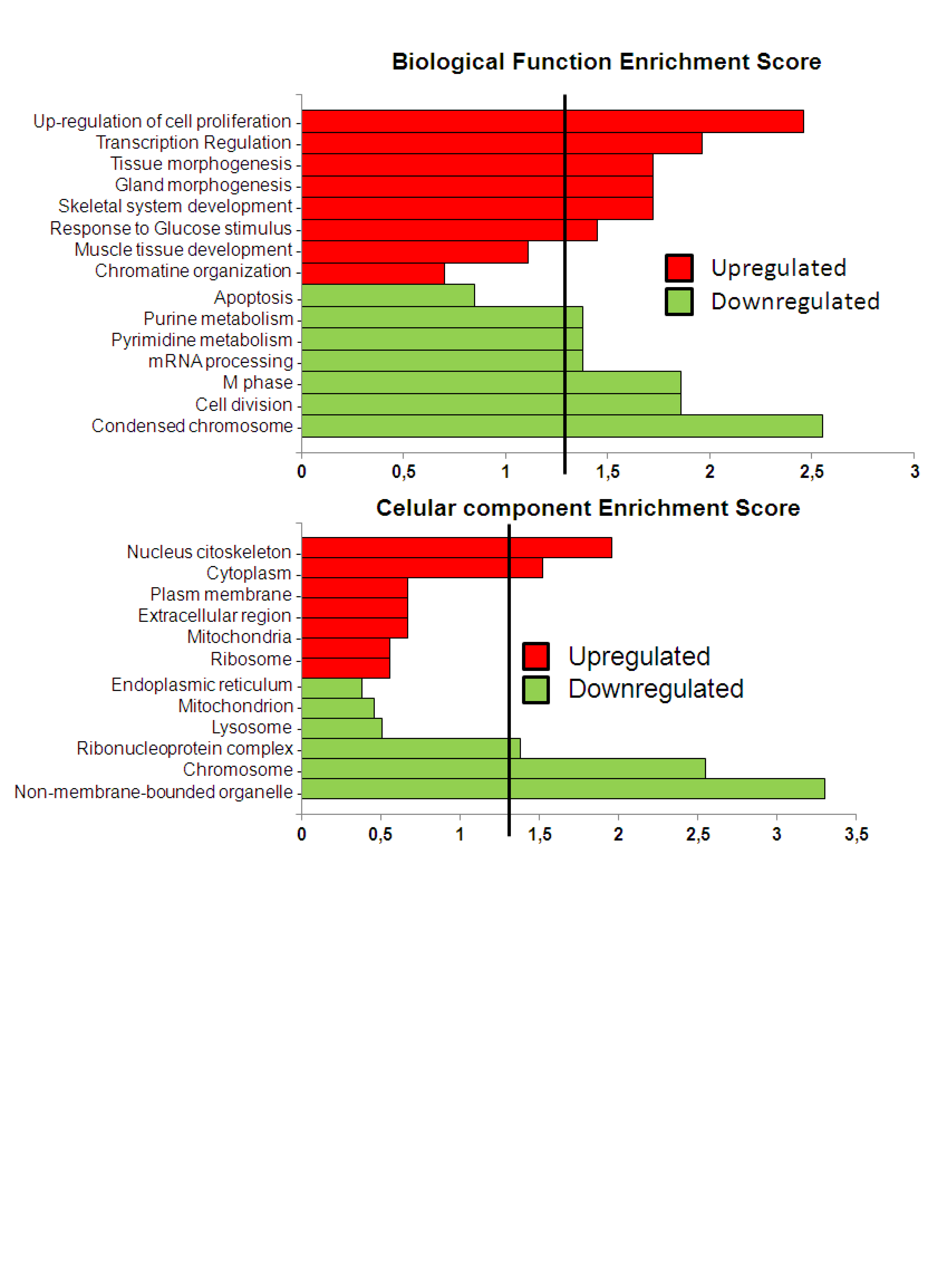

Supplement: Additional file 4: Figure S3 — FatiGo functional analysis of MF vs CF. [file 1471-2164-15-327-S4.tiff]

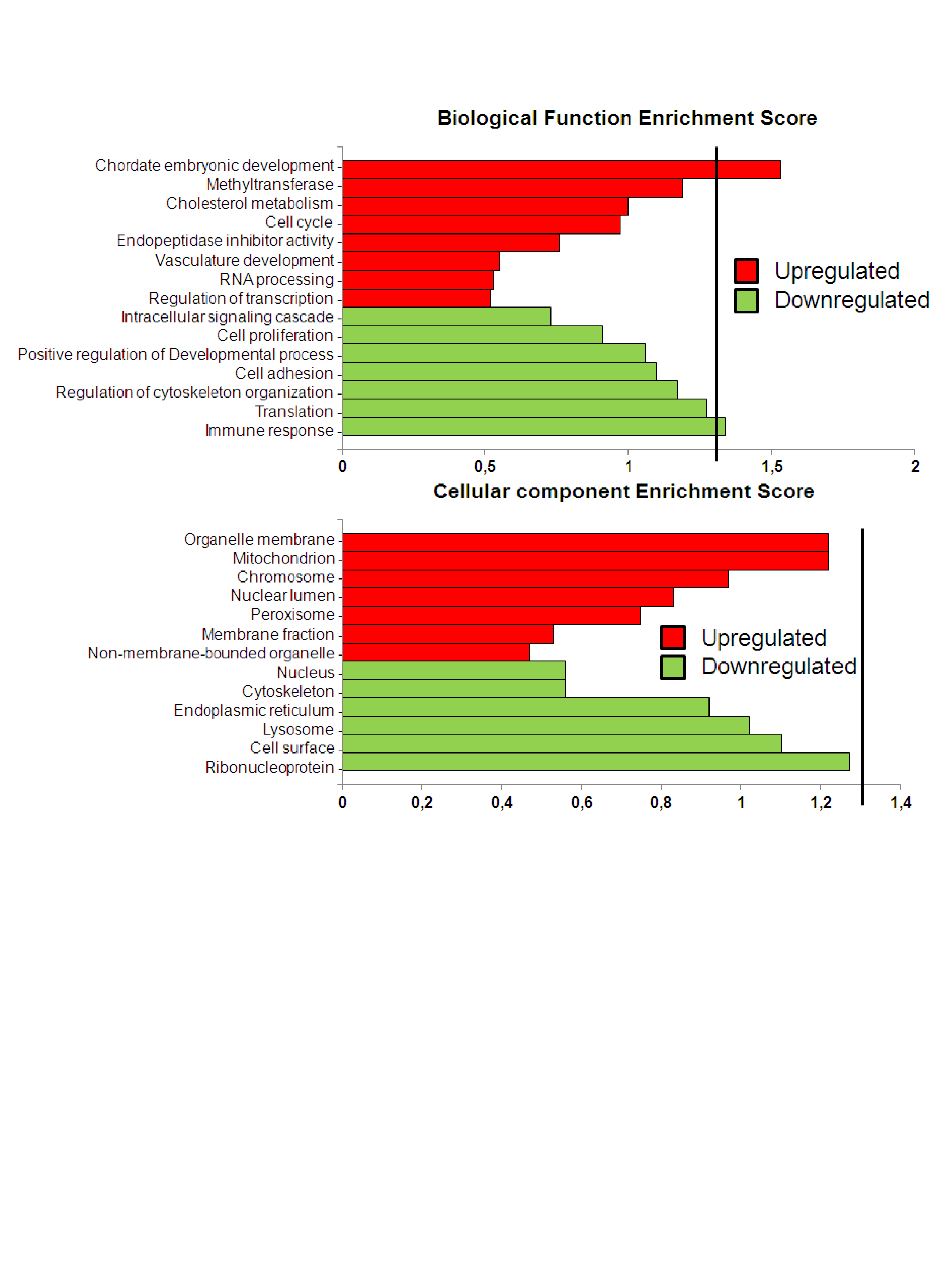

Supplement: Additional file 5: Figure S4 — FatiGo functional analysis of CM vs CF. [file 1471-2164-15-327-S5.tiff]

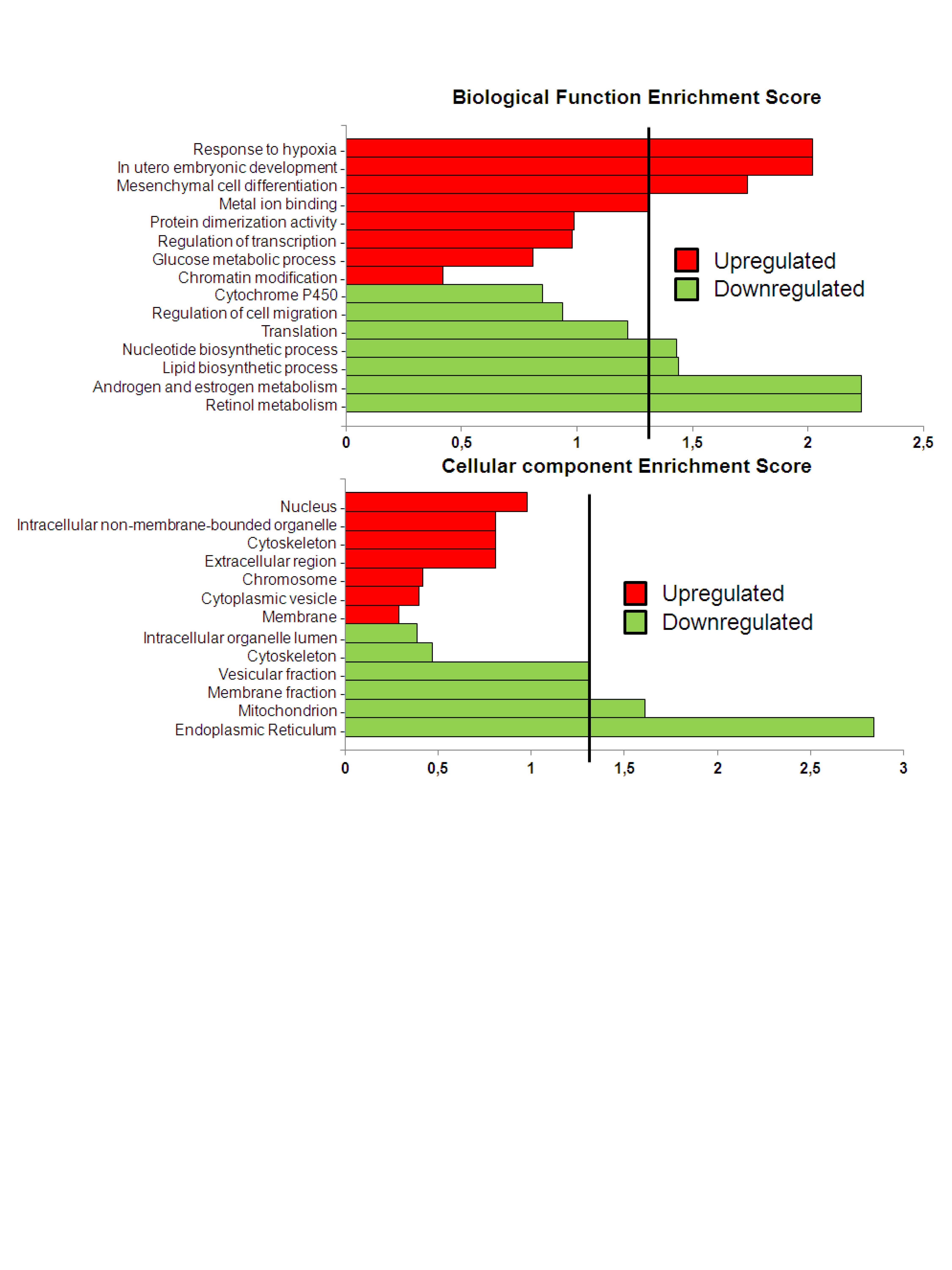

Supplement: Additional file 6: Figure S5 — FatiGo functional analysis of MM vs MF. [file 1471-2164-15-327-S6.tiff]
