# Supplementary material for: Transcriptome profiling of liver of non-genetic low birth weight and long term health consequences
Source: BMC Genomics. 2014 May 1;15:327. doi: 10.1186/1471-2164-15-327 (PMC4229907; doi:10.1186/1471-2164-15-327)
Supplement: Additional file 8: Table S3 — Primers used for RT-PCR. [file 1471-2164-15-327-S8.docx]

**Additional file 8: Table S3.** Primers used for RT-PCR

| **Gene** | **Primer sequence 5´->3´** |
| --- | --- |
| *H2az* | AGGACGACTAGCCATGGACGTGTG/ CCACCACCAGCAATTGTAGCCTTG |
| *Smarcc1* | CAAGAGCCTCTGGGAAGGTG/ CAAGCTTCTCTGGCTCGTCA |
| *Ruvbl2* | GAGTGAGGACGCCTACACAG/ ATGAGCTGGATGGCATAGCG |
| *Hdac5* | TGAGTGCCTGACTGCCTCGC/ GCCTGACATGCCATCTGCCG |
| *Kap1 (Trim28)* | GGAATGGTTGTTCATTGGTG/ ACCTTGGCCCATTTATTGATAAAG |
| *Irs2* | GTACCCTGGGAGGAGGTGAT/ AGCGCTTCACTCTTTCACGA |
| *Gata1* | AAAAGAAGCGGGGGTCGAAT/ TAGGCCCTGATAGAGGTGGG |
| *Igf1* | CGAATGTTCCCCCAGCTGTTT/ GTTTGTCGATAGGGACGGGG |
| *Crabp2* | CTGTGCGAACCACGGAGATT/ GCAGTTCTTGGACCCGTAGG |
| *Chd1* | CTCGATCGCGGTCTTGG/ TCCGCTGCCATTTCTAACACT |
| *Vegfa* | GGGAGTCTGTGCTCTGGGAT/ GGTGTCTGTCTGTCTGTCCG |
| *Fgf23* | TGTGCAATGCTAGGGACCTG/ GTACAGGTGGGTCAGGCTTC |
